# Communication across language barriers in Nordic paediatric oncology care – A cross-sectional multicentre survey with healthcare personnel

**DOI:** 10.1016/j.pecinn.2025.100395

**Published:** 2025-04-30

**Authors:** Melissa Jakobsson, Helena Ventovaara, Johanna Granhagen Jungner, Eva Broström, Elisabet Tiselius, Pernilla Pergert

**Affiliations:** a Department of Women's and Children's Health, Karolinska Institutet, Stockholm, Sweden.; b Department of Swedish Language and Multilingualism, Institute for interpreting and translation studies, Stockholm University, Stockholm, Sweden; c Centre for Research Ethics & Bioethics, Department of Public Health and Caring Sciences, Uppsala University, Uppsala, Sweden.

**Keywords:** Communication, Language barriers, Interpreter, Paediatrics

## Abstract

**Objective:**

The study investigated how healthcare personnel communicate with families when experiencing language barriers, and the use of interpreters in Nordic paediatric oncology care.

**Methods:**

A cross-sectional multicentre survey study with registered nurses (RNs) and medical doctors (MDs) at 20 Nordic paediatric oncology centres. The “Communication over Language Barriers questionnaire” (CoLB-q) was used in the respective Nordic languages. Descriptive analysis and non-parametric tests were used to summarize and compare data.

**Results:**

A total of 489 RNs and MDs completed the survey (response rate of 55 %). Although most respondents reported often or sometimes caring for families with limited proficiency in the country's majority languages, only 20 % had received education in how to use an interpreter. When communicating without an interpreter both professions had used relatives and children as language brokers to some extent. Most respondents assessed that the use of interpreters increased families' participation and improved their care relationships with the families. MDs used interpreters more often than RNs, who in turn used children as language brokers more frequently than the MDs did.

**Conclusion:**

Although most respondents believed that a professional interpreter increases patients' and families' participation and safety in care, children were used as language brokers by both professions. Few had received education or training on how to use an interpreter despite that most participants often met families with limited proficiency in the country's majority language.

**Innovation:**

This study highlights a critical gap and underscores the necessity for healthcare personnel to receive education and training on utilizing professional interpreter resources.

## Highlights

• Language barriers exist in paediatric cancer care and need to be overcome.
• Children are used as language brokers in healthcare.
• Healthcare personnel need education/training in using interpreters.

## 1 Introduction

Immigration to the Nordic countries has increased over the last twenty years, resulting in approximately 3 million foreign born currently living in the Nordic region [1], with varying levels of proficiency in the majority language in their new country. A majority of the immigrants and their descendants in the Nordic countries, live in Sweden [1]. The Nordic countries have shared features in several political areas: one of them is a universal and predominantly publicly financed healthcare [2]. Notably, with globalisation, language barriers have become more prevalent in healthcare settings, and healthcare personnel (HCP) more frequently encounter patients with whom they do not share a common language. Research demonstrates that lack of language skills, in the majority language, is a barrier to healthcare services and contributes to a compromised quality of care [3,4]. Especially for children, language barriers may impact various areas of the delivery and quality of their care [5]. Children whose parents have limited proficiency in the country's majority language have an increased risk for prolonged hospital stay, medical errors, adverse events [6,7], and reduced access to beneficial care [3,8].

Language barriers can be a challenge as they hinder effective communication. HCP are responsible for taking appropriate measures to overcome language barriers in order to provide high-quality care [9,10]. In the Nordic countries, legislations have been established to ensure that patients, understand healthcare information, with Denmark, Iceland, Finland, and Sweden explicitly requiring interpreters [[11], [12], [13], [14]]. The UN Convention on the Rights of the Child guarantees children's right to receive and impart information, participate, and influence matters concerning them [15]. Children should be encouraged to ask questions [16], and parents must receive information and support to participate in their child's care [10,16,17]. An interpreter may be required to uphold these rights [18].

When caring for children, the communication with parents is important in creating a trusting relationship and also to facilitate their understanding of their child's care and treatment [[19], [20], [21]]. The child depends on the parents' ability to understand and comprehend information as well as to communicate the child's needs and wishes [22]. The child's dependence of their parents for communication is even greater in paediatric oncology care, which is a highly specialized field with advanced healthcare information and communication. Accurate information and an honest, clear communication between parents and the HCP are crucial for children's treatment and recovery in paediatric oncology setting. [21,23].

Essentially, it is not language barriers per se that creates risks for negative consequences, but rather the substandard way language barriers are managed by HCP and the insufficient use of language support [5,24,25]. The interpreter's role is important in delivering appropriate care and in improving communication between HCP and families [[24], [25], [26]]. Research shows that interpreters are often underused, and it has been suggested that in order to improve patient-safe care, HCP need to use interpreters more often [24,25,27].

Prior research in Sweden and Denmark has indicated that both siblings and the child itself are often used as language brokers when HCP care for families with limited proficiency in the majority language (LPML), resulting in a heavy responsibility for their own care and treatment [25,28]. When children are used as language brokers, it not only poses risk to the child's clinical course but also disrupts the family relationship. Furthermore, the child is required to translate matters beyond its age and understanding, along with complicated medical terms [29,30]. In a report from the Swedish National Board of Health and Welfare, it has been highlighted that using children as language brokers, endangers patient safety and legal certainty [31]. To our knowledge, there have not been any previous Nordic studies on language barriers in the Nordic countries. To improve the care for children whose parents have limited proficiency in a country's majority language, we need to understand how this type of communication operates in a larger context. Therefore, in this study, our objective was to investigate how HCP communicate with families when experiencing language barriers, and the use of interpreters in Nordic paediatric oncology care.

## 2 Methods

### 2.1 Design

A cross sectional, multicentre survey study.

### 2.2 Instrument

The survey consisted of 10 demographic/background questions followed by the Communication over Language Barriers questionnaire (CoLB-q) [32]. To protect participants' privacy, the number of demographic questions about identifying details were restricted to profession, sex, level of education, length of work experience in paediatrics, and workplace. CoLB-q is a reliable and validated instrument which addresses HCP's experiences regarding communication over language barriers as well as the use of interpreters in paediatric healthcare. The questionnaire consists of 17 questions, 14 closed questions and three open-ended questions. Some of the questions present *Yes* or *No* as response alternatives, while others use a four-point Likert type scale options: *Never, Seldom, Sometimes* and *Often*. One question presents response alternatives *not at all, to a low degree, not so high degree* and *to a high degree*. The CoLB-q was originally developed in Swedish, but has been translated into Finnish, Danish, Icelandic, and Norwegian prior to data collections in those countries.

### 2.3 Setting and participants

Data were collected at all the paediatric oncology centres in Sweden (*n* = 6), Finland (*n* = 5), Denmark (n = 4), Iceland (*n* = 1) and Norway (*n* = 4). Childhood cancer treatment in the Nordic countries is centralized to these centres at public university hospitals, directing and providing the treatment in collaboration with shared care hospitals. All registered nurses (RN), nursing assistants (NA) and medical doctors (MD) who worked in direct patient care, either in inpatient or outpatient units, were invited to participate. However, as there were barely any NAs at the centres apart from Sweden, this group was excluded from analysis. The Swedish NAs experiences have been reported in another study [28].

### 2.4 Data collection

Data collection was carried out between 2016 and 2022. The study was launched in Sweden in 2016, and the data collection in Finland and Denmark started in 2019, in Iceland in 2021, and in Norway in 2022. Data collection was performed by local study coordinators, such as research nurse, consultant RNs or members of the working group on ethics of the Nordic Society of Paediatric Haematology (NOPHO) and the Nordic Society of Paediatric Oncology Nurses (NOBOS). In Sweden, Iceland, and Finland data were collected by using paper questionnaires distributed by local study coordinators, who also collected the completed ones and returned them to the research group by post. In Denmark and Norway web-based questionnaires were used and the link was sent by the local study coordinators to the HCP's professional email addresses. The research team could not see who had answered the questionnaire, except from the demographic information provided.

### 2.5 Data analysis

All statistical analyses were conducted by using IBM SPSS Statistics, version 29. Descriptive statistical analysis was used and presented as frequencies and percentages. Relations between variables were evaluated using cross tabulations and Pearsons Chi-square test. Fisher-Freeman-Halton tests were run when the expected count was less than five in more than 20 % of the cells. Group comparisons were made by evaluating differences between professions (RNs versus MDs), and between countries. Differences between countries were also evaluated by comparing RNs answers. Due to small number of participating MDs, differences between countries were not evaluated by comparing MDs alone. Regarding the analysis of situations when an interpreter is used, respondents who had answered that they *never* communicated with the help of an interpreter were excluded. *P*-values below 0.05 were considered statistically significant.

### 2.6 Ethical considerations

The process of the study was in accordance with the ethical standards of the Declaration of Helsinki and its amendments [33]. In Iceland, the study was approved by the ethical committee of administrative research [11/2021] at the national university hospital of Iceland. In Denmark, Finland, Norway, and Sweden, no ethical review or approval was required. The regional ethical review board in Stockholm had no ethical objections in its advisory statement (2015/1782–31/5). In Finland, research permissions were granted from all five university hospitals. Furthermore, all participants were given written information about the aim of the study, the voluntary nature of participation and that the data would be handled confidentially. In the paper survey, respondents agreed to participate by completing and returning the survey, thereby implying consent. In the online survey, the respondents had to consent before proceeding with the rest of the survey. The questionnaires were anonymous in the sense that they were not provided an identification number connected to the participant. However, since information about sex was collected, for example, the responses from a male nurse could have been tracked. Thus, anonymity was not promised but rather confidentiality. To further ensure confidentiality, participants' answers were combined and presented only on a group level.

## 3 Results

Out of 881 RNs and MDs who were invited to participate, 489 completed the survey (397 RNs and 92 MDs), which corresponds to a 55 % response rate. From this sample, 20 participants were excluded: 12 who did not work in direct patient care, two who had not answered the question about working in direct patient care and six who had not answered any questions at all. 469 participants were included in the analysis, of which 378 were RNs and 91 MDs.

### 3.1 Participants

**Fig. 1:**
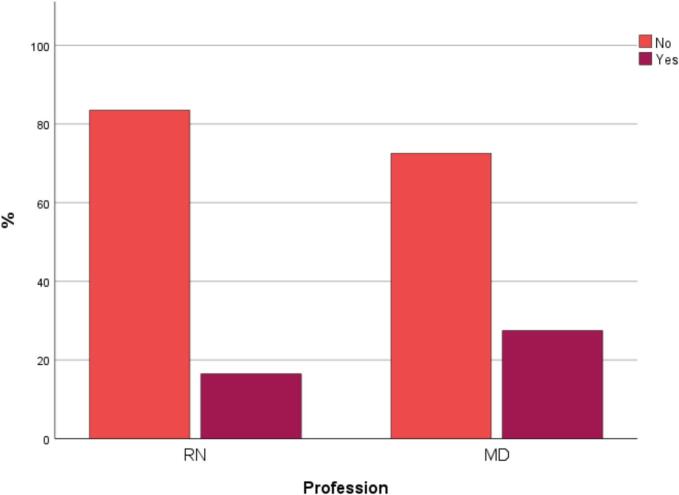
Registered nurses' and medical doctors' answers to the question “Have you received education or training in using an interpreter at work?”, *n* = 467.

**Fig. 2:**
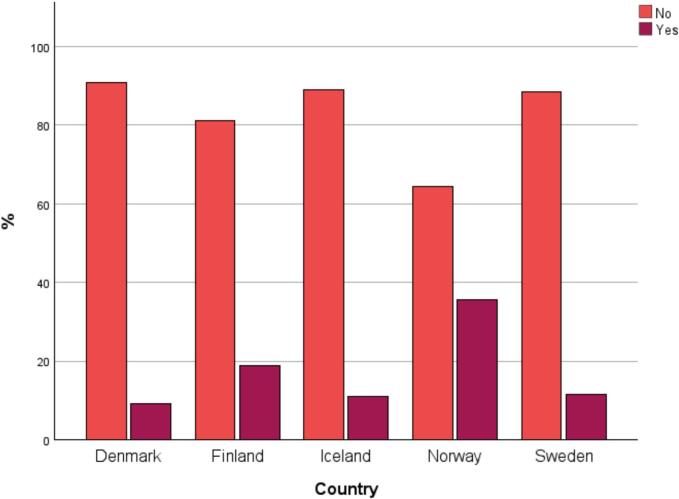
Registered nurses' answers to the question, “Have you received education or training in using an interpreter at work?” split by country, *n* = 376.

Table 1 shows the demographic profile of the participants. The majority were females, and the predominant professional group was RN. Sweden had the largest sample of participants and Iceland the smallest. More than half of the participants reported continued education and over 10 years' working experience in paediatric care. Of all participants, barely 20 % (87/467) reported education or training in using interpreters. As seen in Fig. 1, education in using interpreters was more common among MDs (27 %; 25/91) than among RNs (16 %; 62/376). When comparing RNs across countries, it was most common that RNs in Norway were trained in using interpreters (Fig. 2).

**Table 1:** Overview of the characteristics of participating healthcare personnel.

	Healthcare personnel, n (%)					
Characteristics	All participants	Denmark	Finland	Iceland	Norway	Sweden
**Profession**	**n = 469**	**n = 77**	n **= 99**	n **= 22**	n **= 70**	n **= 201**
Medical doctor	91 (19)	11 (14)	8 (8)	4 (18)	14 (20)	54 (27)
Registered nurse	378 (81)	66 (86)	91 (92)	18 (82)	56 (80)	147 (73)
**Sex**	**n = 467**	n **= 76**	n **= 98**	**n = 22**	**n = 70**	**n = 201**
Male	62 (13)	5 (7)	6 (6)	2 (9)	7 (10)	42 (21)
Female	405 (87)	71 (93)	92 (94)	20 (91)	63 (90)	159 (79)
**Type of patient care**	n **= 466**	n **= 75**	n **= 99**	**n = 22**	**n = 70**	n **= 200**
Inpatient care	297 (64)^a^	35 (47)	80 (81)	21 (95)	47 (67)	114 (57)
Outpatient care	31 (7)^a^	7 (9)	5 (5)	0 (0)	6 (9)	13 (7)
In- and outpatient care	125 (27)^a^	33 (44)	14 (14)	0 (0)	12 (17)	66 (33)
Other	13 (3)^a^	0 (0)	0 (0)	1 (5)	5 (7)	7 (4)
**Continued education**^b^	n **= 463**	n **= 74**	**n = 99**	n **= 21**	n **= 70**	n **= 199**
Yes	275 (59)	31 (42)	62 (63)	9 (43)	38 (54)	135 (68)
No	188 (41)	43 (58)	37 (37)	12 (57)	32 (46)	64 (32)
**Working years in paediatric care**	**n = 467**	n **= 77**	**n = 99**	**n = 22**	n **= 69**	**n = 200**
<1 year	41 (9)^a^	8 (10)	9 (9)	4 (18)	3 (4)	17 (9)^a^
1–2 years	49 (11)^a^	11 (14)	5 (5)	4 (18)	5 (7)	24 (12)^a^
3–4 years	49 (11)^a^	12(16)	7 (7)	4 (18)	10 (15)	16 (8)^a^
5–10 years	84 (18)^a^	12 (16)	16 (16)	3 (14)	18 (26)	35 (18)^a^
>10 years	244 (52)^a^	34 (44)	62 (63)	7 (32)	33 (48)	108 (54)^a^

a Due to rounding error, some of the percentages do not add up to 100 %^.^

b Specialist training or professional continuing education (e.g. paediatrics for nursing assistants, paediatric−/paediatric oncology nursing, paediatric residency).

Nearly all participants reported that they either *often* (50 %; 232/469) or *sometimes* (44 %; 205/469) cared for families and patients with LPML of the country. Only 7 % (32/469) reported caring for patients/families with LPMLs *seldom* and none of the respondents reported *never*.

**Fig. 3:**
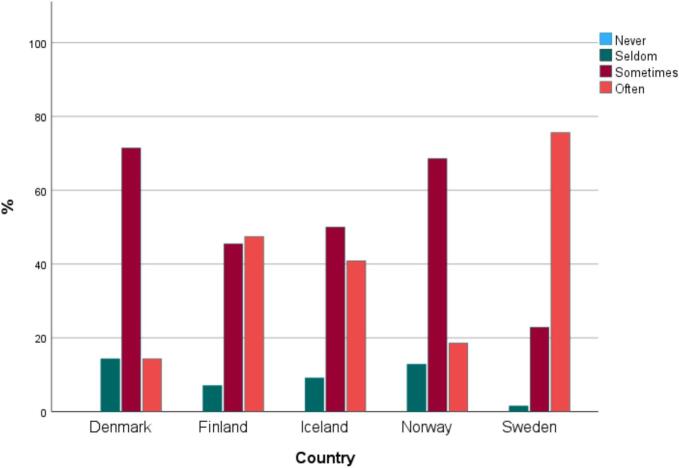
Registered nurses' and medical doctors' answers (*n* = 469) to the question “how often do you care for patients/families who have limited proficiency in the majority language”, split by country.

Caring for families with LPML was most common in Sweden. A majority of Swedish participants reported *often* taking care of families with LPML (76 %; 152/201). In Denmark, Iceland, and Norway, it was more common to *sometimes* do that, as illustrated in Fig. 3.

### 3.2 Communication across language barriers without an interpreter

As seen in Table 2, it was common for both RNs and MDs to use close relatives and family members as language brokers when communicating without a professional interpreter, and to some extent both professions used children (either the patient or a sibling). There were differences between professions: RNs more frequently used children as language brokers.

**Table 2:** Communication without interpreter, split by profession.

	Participants, n (%)		
Statements and response alternatives	RN	MD	*P-*value
**An adult family member/close relative translates, n = 465**			0.301
Never	7 (2)^a^	0 (0)	
Seldom	88 (24)^a^	27 (30)	
Sometimes	204 (55)^a^	50 (55)	
Often	75 (20)^a^	14 (15)	
**A child (e.g., the patient or a sibling) translates, n = 466**			< 0.001
Never	36 (10)^a^	12 (13)^a^	
Seldom	146 (39)^a^	54 (59)^a^	
Sometimes	164 (44)^a^	24 (26)^a^	
Often	29 (8)^a^	1 (1)^a^	
**A colleague translates, n = 459**			0.049
Never	108 (29)	14 (15)	
Seldom	169 (46)	50 (55)	
Sometimes	84 (23)	26 (29)	
Often	7 (2)	1 (1)	
**You speak the language, n = 456**			<0.001
Never	119 (33)^a^	6 (7)^a^	
Seldom	108 (30)^a^	37 (41)^a^	
Sometimes	109 (30)^a^	32 (35)^a^	
Often	29 (8)^a^	16 (18)^a^	

RNs = registered nurses, MDs = medical doctors.

a Due to rounding error, some of the percentages do not add up to 100 %^.^

It was more common among MDs to speak the language in question and vice versa: one third of the RNs (33 %; 119/365) reported that they *never* spoke the language in question, while only 7 % (6/91) of the MDs reported *never.*

Table 3 shows alternative ways of communicating without an interpreter that could be used by RNs in the Nordic countries. Using relatives as language brokers was most common in Sweden, where almost 90 % of the RNs reported that they *often* or *sometimes* communicated with the help of a patient's “family member or a close relative”. In all Nordic countries, RNs used children (patients or siblings) as language brokers to some extent, though there were significant differences between countries. Almost double the percentage of RNs in Sweden and Finland reported *sometimes* or *often* using children as language brokers when communicating without an interpreter compared to other Nordic countries.

**Table 3:** Registered nurses' answers to the question “How often do you communicate without an interpreter in the following ways”, split by countries.

	Registered nurses, n (%)					
	Denmark	Finland	Iceland	Norway	Sweden	*P-*value
**A close relative translates, n = 374**						< 0.001
Never	3 (5)	3 (3)	0 (0)	1 (2)^a^	0 (0)	
Seldom	26 (40)	14 (16)	6 (35)	25 (45)^a^	17 (12)	
Sometimes	32 (49)	55 (62)	9 (53)	28 (50)^a^	80 (54)	
Often	4 (6)	17 (19)	2 (12)	2 (4)^a^	50 (34)	
**A child/sibling translates, n = 375**						< 0.001
Never	13 (20)^a^	4 (5)^a^	2 (12)^a^	4 (7)^a^	13 (9)^a^	
Seldom	32 (49)^a^	22 (25)^a^	9 (53)^a^	35 (63)^a^	48 (33)^a^	
Sometimes	17 (26)^a^	57 (64)^a^	4 (24)^a^	16 (29)^a^	70 (48)^a^	
Often	4 (6)^a^	6 (7)^a^	2 (12)^a^	1 (2)^a^	16 (11)^a^	
**A colleague translates, n = 368**						< 0.001
Never	30 (46)	27 (32)	6 (38)	18 (33)^a^	27 (18)	
Seldom	28 (43)	45 (53)	5 (31)	36 (65)^a^	55 (37)	
Sometimes	5 (8)	12 (14)	5 (31)	1 (2)^a^	61 (42)	
Often	2 (3)	1 (1)	0 (0)	0 (0)^a^	4 (3)	
**You speak the language, n = 365**						< 0.001
Never	22 (34)^a^	15 (18)	1 (6)	13 (23)	68 (47)	
Seldom	20 (31)^a^	24 (29)	6 (35)	28 (50)	30 (21)	
Sometimes	16 (25)^a^	34 (41)	6 (35)	15 (27)	38 (26)	
Often	6 (9)^a^	10 (12)	4 (24)	0 (0)	9 (6)	

a Due to rounding error, some of the percentages do not add up to 100 %^.^

When studying alternative ways that both RNs and MDs used to overcome language barriers, the most used communication tool was a web-based translation tool on a computer, followed by written material (**Supplementary Table 1**). A total of 378 RNs and 91 MDs had answered the open question about other strategies of communicating with the patients and families, and many of them reported using body language or facial expressions, drawing together with the child or using picture communication with material available at the ward.

### 3.3 Communication across language barriers with an interpreter

When communicating with the help of an interpreter, a higher proportion of MDs reported *often* using on-site interpreters (51 %; 46/91) or interpreters over the phone (38 %; 34/90) than did the RNs (24 %; 87/358 resp. 12 %; 42/360). Video interpreting was rarely used. (**Supplementary Table 2**).

Of all participants in the Nordic countries, 27 % (119/449) reported that they *often* used interpreters when informing patients and families about procedures and examinations, and 23 % (104/447) when informing about routines. Only one out of five *often* used interpreters when holding patient/parent education or when preparing for procedures/examinations. When comparing professions, MDs were more likely to use interpreters than the RNs (Table 4).

**Table 4:** Healthcare personnel's answers to the question about how often they perform any of the following with the help of an interpreter” (excluding those who answered never using an interpreter), split by profession.

	Participants, n (%)			
	All	RN	MD	*P-*value
**Take arrival status/medical history, n = 440**				< 0.001
Never	110 (25)	106 (30)	4 (4)	
Seldom	133 (30)	115 (33)	18 (20)	
Sometimes	126 (29)	92(26)	34 (38)	
Often	71 (16)	38 (11)	33 (37)	
**Inform about routines, n = 447**				0.314
Never	26 (6)	23 (6)^a^	3 (3)	
Seldom	112 (25)	94 (26)^a^	18 (20)	
Sometimes	205 (46)	161 (45)^a^	44 (49)	
Often	104 (23)	79 (22)^a^	25 (28)	
**Inform about procedures/examinations,n = 449**				0.002
Never	11 (2)^a^	10 (3)^a^	1 (1)	
Seldom	92 (20)^a^	82 (23)^a^	10 (11)	
sometimes	227 (51)^a^	185 (52)^a^	42 (47)	
Often	119 (27)^a^	82 (23)^a^	37 (41)	
**Prepare for procedures/examinations, n = 445**				0.731
Never	27 (6)	22 (6)	5 (6)	
Seldom	141 (32)	116 (33)	25 (28)	
Sometimes	193 (43)	153 (43)	40 (44)	
Often	84 (19)	64 (18)	20 (22)	
**Hold patient/parent education, n = 443**				0.371
Never	39 (9)^a^	30 (8)	9 (10)	
Seldom	136 (31)^a^	114 (32)	22 (25)	
Sometimes	177 (40)^a^	143 (40)	34 (39)	
Often	91 (21)^a^	68 (19)	23 (26)	
**Have supportive conversations, n = 444**				< 0.001
Never	55 (12)	52 (15)^a^	3 (3)	
Seldom	162 (36)	147 (42)^a^	15 (17)	
Sometimes	174 (39)	123 (35)^a^	51 (57)	
Often	53 (12)	32 (9)^a^	21 (23)	
**Smalltalk, n = 443**				0.104
Never	228 (51)^a^	186 (53)	42 (47)	
Seldom	163 (37)^a^	132 (37)	31 (35)	
Sometimes	44 (10) ^a^	29 (8)	15 (17)	
Often	8 (2)^a^	7 (2)	1 (1)	

RNs = registered nurses, MDs = medical doctors.

a Due to rounding error, some of the percentages do not add up to 100 %^.^

When comparing the use of interpreters between countries solely among RNs, there were differences in how often they were used. Approximately 30 % of RNs in Sweden and Finland reported that they used interpreters *often* when they informed about routines or procedures and examinations, while in other Nordic countries 10 % or less did that *often*. It was most common in Finland to *often* use interpreters for holding patient education (41 %; 34/84), followed by Sweden where 14 % (20/140) reported *often* using interpreters for that purpose.

### 3.4 HCP's experiences of the impact of interpreter use on the families' and children's care

Most RNs and MDs assessed that the use of interpreters increased patients' and families' participation and safety in care *to a high degree*. Similarly, most participants assessed that the use of interpreters improved their care relationships with patients and families *to a high degree*.

**Fig. 4:**
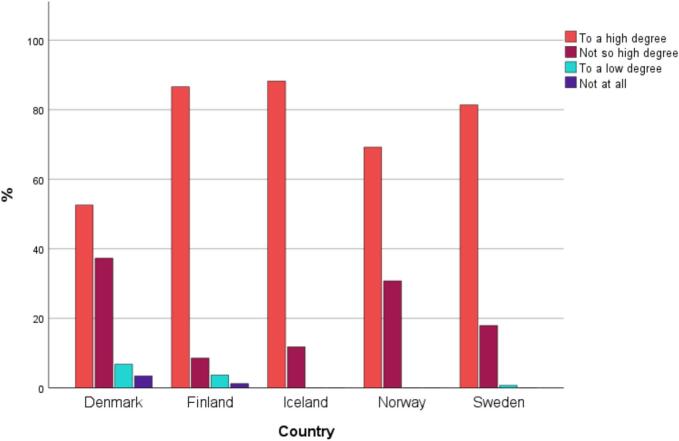
Registered nurses' answers (*n* = 355) to the question regarding if they thought that the use of interpreters “Improves your health care relationship with the patient/family”, split by countries.

Among RNs alone, the vast majority answered that the use of interpreters increased patients' and families' participation in care “*to a high degree*”. However, only 53 % (31/59) of the Danish RNs believed that the use of interpreters improved their care relationships with the patients and families *to a high degree*, while 69 to 88 % of RNs in the other Nordic countries reported so (Fig. 4).

## 4 Discussion and conclusion

### 4.1 Discussion

This study investigated how HCP communicate with families when experiencing language barriers, and the use of interpreters in Nordic paediatric oncology care. While the participants in this study often cared for families with LPML, most of them did not have education or training in using interpreters. The results show that both professions to some extent used family members and children as language brokers, and that in general, interpreters were rarely used for patient education or when preparing children and families for procedures or examinations. However, there were differences between professions and between countries.

In the Nordic countries, nearly all HCP reported *often* or *sometimes* caring for families with LPMLs. This aligns with previous research from Denmark, where 56 % of HCP reported encountering language barriers with families in the paediatric emergency department on a weekly basis [25]. Furthermore, our study shows that RNs in Sweden meet families with LPML more often than RNs in the rest of the Nordic countries. This result is somewhat expected since Sweden has had the largest immigration among the Nordic countries [34]. These findings confirm that paediatric patients with families with LPML are a common patient group within the Nordic healthcare setting and emphasizes the importance of improved communication when caring for these patients and their families. One could argue that a high concentration of patients with language barriers should lead to comparable legislation and institutional policies across the Nordic region to address these barriers.

Furthermore, education or training in the use of interpreters was overall rare among HCP. A similar finding was reported by Dungu et al. [25], who found that only 12 % of HCP had received training in the use of interpreters. The importance of training has been highlighted in previous studies: it allows the evaluation of the interpreter's ability to correctly interpret as well as the optimal use of interpreter services [[35], [36], [37]]. Our results show that education/training in using interpreters was most common among MDs. Prior studies suggest that training and education in using interpreters should be implemented in hospitals for all HCP [25,[38], [39], [40]]. Furthermore, a study from Sweden [41] showed that none of the participating RNs had received any education in utilizing translation services. The RNs in the Swedish study wished for training in the utilization of interpreters and suggested that such training should be implemented in the work organization or the nursing education [41]. Results from other studies also show that HCP want a better understanding about regulations regarding the utilization of interpreters, as to who decides when an interpreter is needed [38,39], as well as a higher awareness of the existing interpreter-related guidelines in the healthcare system [25,40].

Moreover, in our study, MDs and RNs primarily used interpreters on-site, followed by telephone interpreters, and rarely through video. The differences in the methods of communication through an interpreter may not, however, be completely accurate as data was collected at different points in time in the participating countries. For instance, the use of technology for interpretation by HCP may have been affected by the Covid-19 pandemic. Keller et al. [27] describe a switch in the health care setting during the Covid-19 pandemic, where, for example, virtual interpretation services increased. Nevertheless, research indicates that the means for interpretation services may not be as important as the fact that a professional interpreter is used [26]. Another finding was that MDs were using interpretation services more frequently than RNs, which also has been reported in previous studies [28,40]. This may be due to that MDs communicate information of a more medical and complex nature, judged to be more important or difficult, and therefore in a greater need of professional interpretation. Although interpretation services were used more frequently by MDs, our study also showed that other communication tools were used by all HCP. Essentially, this finding may indicate that MDs communicate in a more formal setting whereas RNs rely on bedside communication with the families. This suggests a need for solid and reliable communication tools available to RNs when having communication with families with LPML.

Regarding communication without an interpreter, our study found that family members and children were used as language brokers by both professions to some extent. It is confirmed by other studies that children are used as language brokers within the healthcare system [4,25,27,28]. This becomes problematic as children are not developmentally mature enough to act as language brokers in the healthcare setting and thus potentially risks the dynamics within the family [18,30]. The use of children as language brokers raises ethical concerns as they may be exposed to distressing information regarding their own or a family member's health and prognosis [18,29,30]. There is also a risk that children might withhold or alter information for the sake of family members or to handle sensitive matters [18]. Furthermore, we would argue that there is a risk of obtaining informed consent that does not meet accepted standards and that parental involvement in decision-making is undermined. This could potentially cause misunderstandings of the risks, benefits and options of participation in research, medical procedures and treatments, which could lead to ethical and legal issues.

The consequences of mistakes or wrong translations can be serious. A professional interpreter is therefore essential to achieve an accurate communication and to reduce the risk of clinically important medical errors [42]. When further investigating the use of children as language brokers in our study, we found differences between countries. A higher proportion of RNs in Sweden and Finland used children as language brokers more often compared to the RNs in the other Nordic countries. These differences may be explained by different legislations where for example Norway has introduced legislation that obliges government and HCP to use professional interpreters in case of language barriers. Furthermore, it is illegal in Norway to use children as language brokers in contact with authorities [43]. When comparing RNs with MDs, we also found that it was more common that RNs used children as language brokers. Similar results have been shown in previous research where it was found more common to use professional interpreters to describe a medical treatment, while information regarding nursing care was often communicated via non-professional interpreters instead [40].

Another objective of our study was to determine to what extent interpreters were used in different communication situations in healthcare. One finding was that only 27 % of all respondents often used interpreters when informing patients and families about procedures and examinations. In accordance with this, a US study demonstrated a particularly low level of interpreter use in high-risk activities in the paediatric emergency department, such as the administration of medication and procedures [44]. Furthermore, Dungu et al. [25] found that HCP experienced difficulties to provide relevant information and instructions to families when a language barrier was present. The low level of interpreter use in our study when informing about procedures or examinations could therefore be considered surprising, as the study was conducted in paediatric oncology care, a highly specialized healthcare setting [[21], [22], [23]]. If interpreters are rarely used when preparing patients and families for procedures and examinations in this specialized context, one may wonder how often they are used in less specialized or acute care settings. This under use of interpreters is an important aspect as children have a right to information and preparation appropriate to their age and level of development while being in hospital care [16,45]. Lack of preparation before an examination or a procedure may result in feelings of stress and distrust and affect the child's sentiments of being seen and involved [46]. The international collaborative standards to support paediatric patients [16] emphasize that when undergoing healthcare procedures, children should be encouraged to communicate freely and to receive sufficient information, which should not be affected by their own or their parents' language proficiency [16]. Therefore, it could be argued that using an interpreter when preparing families and children for procedures is essential to ensure that children's well-being and rights, as well as patient safety, are not overlooked due to a lack of language skills.

### 4.2 Strengths and limitations of the study

The instrument CoLB-q could be considered a strength of the study as it was developed and evaluated in the paediatric healthcare context [32]. The study's international perspective with a relatively large number of participants may also be considered a strength, increasing the applicability of the findings on a more global level. Nonetheless, one limitation could be the uneven distribution of participants across countries: there were fewer participants from the other Nordic countries than Sweden. However, Sweden has more inhabitants compared to the other Nordic countries. The long data collection period is noteworthy, as the Covid-19 pandemic and technological advances have significantly influenced our communication methods, potentially affecting results such as the frequency of technical device usage. One could also argue that a reasonable question to add to the instrument would be in which specific situations children are used as language brokers, and how HCP obtain valid informed consent despite language barriers. To further deepen the understanding and strengthen the interpretation of the results, on-site observations of communication situations and qualitative interviews are needed, focusing on the child's and the parents' perspectives and their participation in the communication situation. Also, more knowledge is needed about obtaining informed consent and assent to research from families with LPML.

### 4.3 Innovation

This study is innovative in its comprehensive approach to understanding the communication challenges faced by healthcare personnel in Nordic paediatric oncology care. By utilizing a cross-sectional multicentre survey, the research captures a wide range of experiences from both RNs and MDs across 20 centres. This broad scope allows for a more nuanced understanding of how language barriers impact care and the varied use of interpreters. The use of the CoLB-q in multiple Nordic languages further enhances the study's relevance and applicability across different linguistic contexts within the Nordic region.

Moreover, the study highlights a critical gap in the training of healthcare personnel regarding the use of interpreters, with only 20 % of participants having received such education. This finding underscores the need for targeted educational interventions to improve communication in paediatric oncology settings. The innovative aspect lies in not only identifying the problem but also in providing a clear direction for future improvements in healthcare communication practices such as education in interpreter mediated communication among healthcare personnel. By comparing the practices of RNs and MDs, the study offers valuable insights into professional differences and commonalities, paving the way for more effective and inclusive communication strategies in paediatric healthcare.

### 4.4 Conclusion

The results of this study indicate that families with LPML are a commonly occurring patient group within Nordic paediatric healthcare. The study also shows that the interpreter is perceived as important for the family's participation in care as well as the relationship between HCP and the family. However, there are regularly occurring situations where an interpreter is not used, which can jeopardize the provision of patient safe care. Still, what may be considered the most important result of this study is that children (i.e. minors under 18) are used as language brokers within healthcare services. Many countries do not have an explicit prohibition against using children as language brokers, however, according to prior studies it is not compatible with the best interest of the child. The healthcare system should therefore be organized so that the occurrence of children being used as language brokers is as minimized as possible. HCP may need more knowledge and understanding about why an interpreter should be used, and a need for clarification of the children's situation when they are used as language brokers.

## CRediT authorship contribution statement

**Melissa Jakobsson:** Writing – review & editing, Writing – original draft, Visualization, Formal analysis. **Helena Ventovaara:** Writing – review & editing, Writing – original draft, Visualization, Supervision, Formal analysis. **Johanna Granhagen Jungner:** Writing – review & editing, Supervision, Funding acquisition, Data curation, Conceptualization. **Eva Broström:** Writing – review & editing, Funding acquisition. **Elisabet Tiselius:** Writing – review & editing, Supervision, Funding acquisition, Data curation, Conceptualization. **Pernilla Pergert:** Writing – review & editing, Supervision, Resources, Funding acquisition, Data curation, Conceptualization.

## Funding

This work was supported by the Mälardalen Area Research School in Healthcare Science [grant numbers FUS-2023:0004]; and 10.13039/501100004047Karolinska Institutet. The last author was supported by the Swedish Childhood Cancer Fund [TJ2017–0011], including this research. The funders had no role in the design and conduct of the study or in the manuscript process.

## Declaration of competing interest

The authors declare that they have no known competing financial interests or personal relationships that could have appeared to influence the work reported in this paper.

## Data Availability

Data will be made available upon reasonable request.

## Appendix A Supplementary data

Supplementary material Image 1
