# Supplementary material for: Communication across language barriers in Nordic paediatric oncology care – A cross-sectional multicentre survey with healthcare personnel
Source: PEC Innov. 2025 Apr 30;6:100395. doi: 10.1016/j.pecinn.2025.100395 (PMC12098145; doi:10.1016/j.pecinn.2025.100395)
Supplement: Supplementary file 1 — Supplementary material [file mmc1.docx]

**Communication across language barriers without an interpreter**

**Supplementary Table 1. Alternative ways that Registered Nurses (RNs) and Medical Doctors (MDs) used to overcome language barriers without an interpreter.**

|  | **All participants**  **n =463**  **n (%)** | **RN**  **n = 372**  **n (%)** | **MD**  **n = 91**  **n (%)** |
| --- | --- | --- | --- |
|  |  |  |  |
| **Communication through written information** |  |  |  |
| Never | 130 (28) | 95 (26) | 35 (38) |
| Seldom | 188 (41) | 146 (39) | 42 (46) |
| Sometimes | 129 (28) | 117 (31) | 12 (13) |
| Often | 16 (3) | 14 (4) | 2 (2) |
|  |  |  |  |
| **Communication through computer** |  |  |  |
| Never | 104 (22) | 63 (17) | 41 (45) |
| Seldom | 189 (41) | 151 (41) | 38 (42) |
| Sometimes | 141 (30) | 131 (35) | 10 (11) |
| Often | 29 (6) | 27 (7) | 2 (2) |
|  |  |  |  |
| **Communication through app** |  |  |  |
| Never | 199 (43) | 145 (39) | 54 (59) |
| Seldom | 166 (36) | 137 (37) | 29 (32) |
| Sometimes | 84 (18) | 77 (21) | 7 (8) |
| Often | 14 (3) | 13 (3) | 1 (1) |

RNs = registered nurses, MDs = medical doctors.

**Communication ACROSS language barriers with an interpreter**

**Supplementary Table 2. Use of interpreters, on site, via telephone and via video, split by profession.**

|  | RN | | MD | |
| --- | --- | --- | --- | --- |
|  | n (%) |  | n (%) |  |
| **On site, n = 449** |  |  |  |  |
| Never | 5 (1) |  | 0 |  |
| Seldom | 76 (21) |  | 10 (11) |  |
| Sometimes | 190 (53) |  | 35 (38) |  |
| Often | 87 (24) |  | 46 (51) |  |
|  |  |  |  |  |
| **Via telephone, n = 450** | |  |  |  |
| Never | 37 (10) |  | 1 (1) |  |
| Seldom | 110 (31) |  | 15 (17) |  |
| Sometimes | 171 (48) |  | 40 (44) |  |
| Often | 42 (12) |  | 34 (38) |  |
|  |  |  |  |  |
| **Via video, n = 435** |  |  |  |  |
| Never | 272 (78) |  | 71(83) |  |
| Seldom | 61 (17) |  | 13 (15) |  |
| sometimes | 14 (4) |  | 2 (2) |  |
| Often | 2 (1) |  | 0 (0) |  |

RNs = registered nurses, MDs = medical doctors.
